# Non-invasive imaging of atherosclerotic plaque macrophage in a rabbit model with F-18 FDG PET: a histopathological correlation

**DOI:** 10.1186/1471-2385-6-3

**Published:** 2006-05-25

**Authors:** Zhuangyu Zhang, Josef Machac, Gerard Helft, Stephen G Worthley, Cheuk Tang, Azfar G Zaman, Oswaldo J Rodriguez, Monte S Buchsbaum, Valentin Fuster, Juan J Badimon

**Affiliations:** 1 Division of Nuclear Medicine, Department of Radiology, The Mount Sinai School of Medicine, New York, NY, USA; 2 Zena and Michael A. Wiener Cardiovascular Institute, The Mount Sinai School of Medicine, New York, NY, USA; 3 Neuroscience PET Laboratory, Department of Psychiatry, The Mount Sinai School of Medicine, New York, NY, USA; 4 Department of Radiology, The Mount Sinai School of Medicine, New York, NY, USA

## Abstract

**Background:**

Coronary atherosclerosis and its thrombotic complications are the major cause of mortality and morbidity throughout the industrialized world. Thrombosis on disrupted atherosclerotic plaques plays a key role in the onset of acute coronary syndromes. Macrophages density is one of the most critical compositions of plaque in both plaque vulnerability and thrombogenicity upon rupture. It has been shown that macrophages have a high uptake of ^18^F-FDG (FDG). We studied the correlation of FDG uptake with histopathological macrophage accumulation in atherosclerotic plaques in a rabbit model.

**Methods:**

Atherosclerosis was induced in rabbits (n = 6) by a combination of atherogenic diet and balloon denudation of the aorta. PET imaging was performed at baseline and 2 months after atherogenic diet and coregistered with magnetic resonance (MR) imaging. Normal (n = 3) rabbits served as controls. FDG uptake by the thoracic aorta was expressed as concentration (μCi/ml) and the ratio of aortic uptake-to-blood radioactivity. FDG uptake and RAM-11 antibody positive areas were analyzed in descending aorta.

**Results:**

Atherosclerotic aortas showed significantly higher uptake of FDG than normal aortas. The correlation of aortic FDG uptake with macrophage areas assessed by histopathology was statistically significant although it was not high (r = 0.48, p < 0.0001). When uptake was expressed as the ratio of aortic uptake-to-blood activity, it correlated better (r = 0.80, p < 0.0001) with the macrophage areas, due to the correction for residual blood FDG activity.

**Conclusion:**

PET FDG activity correlated with macrophage content within aortic atherosclerosis. This imaging approach might serve as a useful non-invasive imaging technique and potentially permit monitoring of relative changes in inflammation within the atherosclerotic lesion.

## Background

Atherothrombosis, characterized by atherosclerotic lesion disruption with superimposed thrombosis, is the main cause of acute coronary syndromes (unstable angina, myocardial infarction and sudden death) [1]. It represents the major cause of morbidity and mortality in the industrialized world. Experimental, pathological and clinical studies have clearly demonstrated the heterogeneity of atherosclerotic lesions [2]. Typically, the mature atherosclerotic plaque contains two different components: soft lipid/macrophage-rich "atheromatous" material and hard smooth muscle cell-related sclerotic tissue. Significant advances have been made in understanding of mechanisms underlying this disease process. The progression of atherosclerotic plaques in coronary circulation is dependent on a number of risk factors. It has been shown that the atherosclerotic plaque composition rather than the degree of arterial stenosis appears to be a critical determinant of atherosclerotic plaque vulnerability and thrombogenicity [1,3]. The current gold standard imaging technique for atherosclerosis is x-ray contrast angiography, which provides high-resolution definition of the site and severity of luminal stenoses, but no information about plaque composition. Thus other than ulcerated lesions, it cannot differentiate between unstable and stable plaques and, therefore, is unable to predict the risk of plaque rupture. In order to assess the presence, extent and composition of atherosclerotic lesions in patients, there is a clinical need for a non-invasive diagnostic imaging technique which can be used to assess the vulnerability of atherosclerotic plaques [4]. It was reported that macrophages contribute extensively to the development of inflammation in plaques [5,6] and ruptured plaques have large numbers of macrophages [7]. Several pathologic studies have clearly demonstrated that macrophages play a key role in both risk of plaque rupture and modulation of the plaque's subsequent thrombogenicity [3,8]. Tissue factor present in lipid-rich atherosclerotic plaques is important in acute arterial thrombosis and correlates with the extent of areas of macrophages [8,9]. Thus, macrophage density is considered an important determinant of plaque vulnerability. It has been shown that macrophages of tumor tissue have a high uptake of ^18^F-FDG (FDG) [10]. This discovery has led to investigation of the possibility of using FDG to image macrophages in atherosclerotic plaque. The objective of this study was to investigate the feasibility of using FDG as a marker and using PET to assess the macrophage burden in atherosclerotic lesions, and thereby to predict their vulnerability.

## Methods

### Animal model of experimental atherosclerosis

The animal model selected for this study was New Zealand white rabbit (n = 9, 3.0 to 3.4 kg). Atherosclerotic lesions were induced in six rabbits by a combination of a moderate atherogenic diet (0.2% cholesterol-enriched diet, Research Diet, Inc., New Brunswick, NJ, USA) for two months and balloon denudation of the aorta one week after diet initiation [11]. Three normal rabbits with normal diet and without balloon denudation of the aorta served as controls. All procedures were performed under general anesthesia by intramuscular injection of ketamine (20 mg/kg) and xylazine (10 mg/kg). Aortic denudation of the descending thoracic aorta was performed by withdrawal, with moderate resistance, of a 4Fr Fogarty embolectomy catheter introduced through the iliac artery and passed into the aortic arch. Catheter insertion and inflation were repeated for four passes, after which the catheter was removed, the femoral artery tied, and the incision closed. All experiments were approved by the Mount Sinai School of Medicine animal management program, under accreditation from the American Association for the Accreditation of Laboratory Animal Care (AALAC).

### FDG PET imaging

After fasting the previous night, the rabbits were anesthetized as described above. They were immobilized with a body-fitting thermosetting plastic holder compatible with both MR and PET scanning systems, thus assuring the same positioning of the rabbits for both imaging studies. Immediately before the FDG PET imaging, all rabbits underwent MR imaging for the purpose of localization of the anatomical structures on the PET images. The rabbits were laser-aligned on the xiphi-sternum in both scanning modalities. After initial gradient echo scout images to identify the thoracic aorta, 2D-time of flight MR imaging was performed to localize the aorta (field of view: 18 × 11.25 cm; matrix 256 × 160; TR/TE: 46/4.4 msec, slice thickness 2 mm; flip angle: 45°). The anesthetized rabbits were then immediately taken to the PET scanner in the holders. Imaging was performed using a GE 2048-plus brain-dedicated PET scanner. Each rabbit was injected via a marginal ear vein with 55.5 ± 7.4 MBq (1.5 ± 0.2 mCi) of FDG. PET imaging of aorta was performed 30 min post injection. Following transmission (30 min) and emission (3 ten min images) acquisitions, the data were reconstructed with standard filtered back projection and attenuation correction. Using an in-house software system, each PET slice was co-registered with its corresponding MR imaging slice [12]. Thus, anatomical structures identified by MR imaging were correlated with FDG images by a process of image coregistration.

### Blood sampling

Arterial blood samples were collected from an auricular artery at 15, 30, 45, and 60 min post-injection of FDG. Blood samples were weighed. The radioactivity was counted in a γ counter (Minaxi Auto-Gamma 5000 Series Gamma Counter, Packard, USA) and decay corrected.

### Histopathology

To correlate the PET data with histopathological observations, rabbits were euthanized within 24 hours of PET imaging by intravenous injection of 5 mL "Sleepaway" IV (Fort Dodge Animal Health, Fort Dodge, IA) after administration of heparin (100 IU/kg) to prevent post-mortem blood clotting. The aortas were immediately flushed with 250 ml of physiological buffer (0.1 mol/L PBS, pH 7.4) followed by perfusion fixation with 250 ml cold (4°C) 4% paraformaldehyde in 0.1% PBS. Perfusions were performed at 100 mmHg. The entire aorta from the aortic root to the iliac arteries was excised. After perfusion fixation, all specimens were immersed in fresh fixative and stored at 4°C. Serial sections of the aorta were cut at 6 mm intervals, matching corresponding MR-PET images. Coregistration was carefully performed, based on the position of the aortic arch. Aortic specimens were embedded in paraffin and sections 5-micron thick were cut. The extent of atherosclerosis burden was confirmed by CME staining macrophage content. Immunohistology was performed using a monoclonal antibody directed against macrophages (RAM-11) to assess macrophage content. RAM-11 antibody (Dako North America, Inc., Carpinteria, CA, USA) was diluted 1:1000 and visualized using Horseradish Peroxidase ABC kit (Vector Laboratories, Burlingame, CA, USA) as per manufacturer's instructions. RAM-11 macrophage positive areas were measured using a computer-assisted quantitative color image analysis system (Image ProPlus, Media Cybernetics) and expressed in mm^2^.

### Data analysis

Regional analyses of MR-PET images were performed and related this information to corresponding regional macrophage areas. The PET images were transferred to a PC compatible computer for further analysis. For the definition of regions of interest (ROI) and data analysis, in-house computer programs were used. ROIs of the aorta identified by MR imaging were correlated with FDG PET images of the same animal by image coregistration. Aortic uptake, which was expressed as concentration (μCi/ml), was measured for the eight consecutive upper sections of the descending thoracic aorta (length 4.8 cm). Slices were 6 mm apart by taking a 5 × 5 mm^2 ^ROI. For the correction of blood pool activity, FDG uptake was also expressed as the ratio of aortic uptake-to-blood radioactivity. The histopathological sections were digitized to a Macintosh computer from a Sony 3CCD Video camera attached to a Zeiss Axioskop light microscope. RAM-11 macrophage positive areas were measured using a computer-assisted quantitative color image analysis system (Image ProPlus, Media Cybernetics) and expressed in mm^2^. FDG uptake on each slice was correlated to the corresponding histological results.

### Statistical analysis

The correlations between regional measurements by PET and histopathology on the slices (n = 72) of descending thoracic aorta of the rabbits were analyzed by simple linear regression with 95% confidence intervals (Statview, SAS Institute Inc., Cary, NC, USA). A p value < 0.05 was considered statistically significant.

## Results

### Atherosclerotic characterization by histology

**Figure 1:**
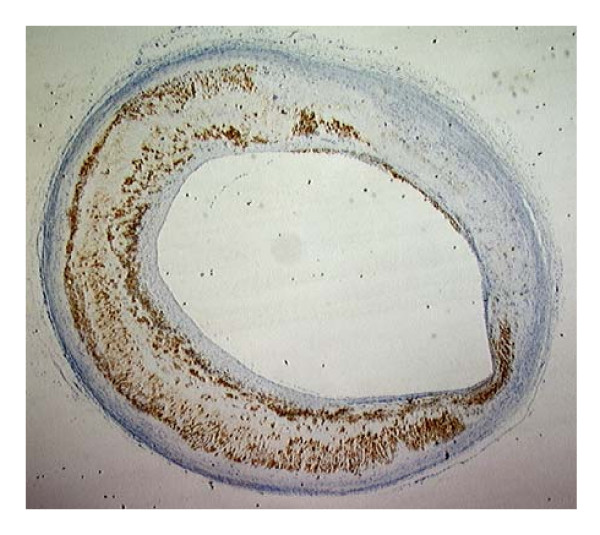
Atherosclerotic Rabbit Aorta: RAM-11 staining.

In the control group, there were no atherosclerotic lesions and no macrophages in the aortic wall. The combination of an atherogenic diet and balloon denudation of the aorta induced significant atherosclerosis. The induced atherosclerotic lesions contained macrophages as indicated by RAM-11 staining (Figure 1).

### FDG PET imaging and coregistration of MRI and PET images

**Figure 2:**
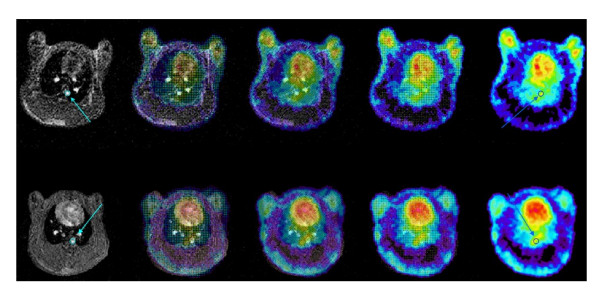
**Coregistration of MRI and PET images**. Coregistration of MRI and FDG PET images of a control (top) and an atherosclerotic rabbit (bottom). Each row shows the fused dataset progressing from an anatomical MRI to a sole functional FDG PET image of the same animal in the same location. ROIs of the aorta (indicated with a blue circle and arrow) were identified on the MRI. The software automatically extracted the PET values from the same location in the PET image.

**Figure 3:**
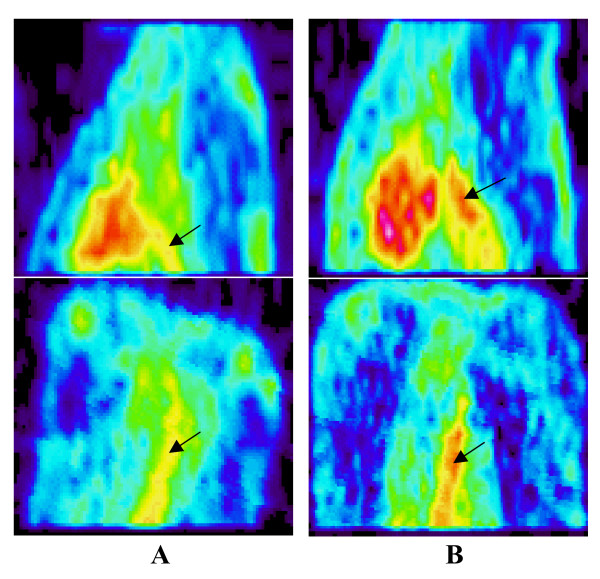
***In vivo *PET images**. *In vivo *PET sagittal (top) and coronal (bottom) images showing the uptake of FDG in the thoracic aorta in a control rabbit (A), in a rabbit with mild atherosclerosis (B).

ROIs of the aorta identified by MR imaging were correlated with FDG PET images of the same animal by image coregistration (Figure 2). In FDG PET images, sagittal and coronal sections of the descending aorta of atherosclerotic rabbits (Figure 3B) demonstrated a higher uptake of FDG than controls (Figure 3A).

### Macrophage areas and FDG uptake

**Figure 4:**
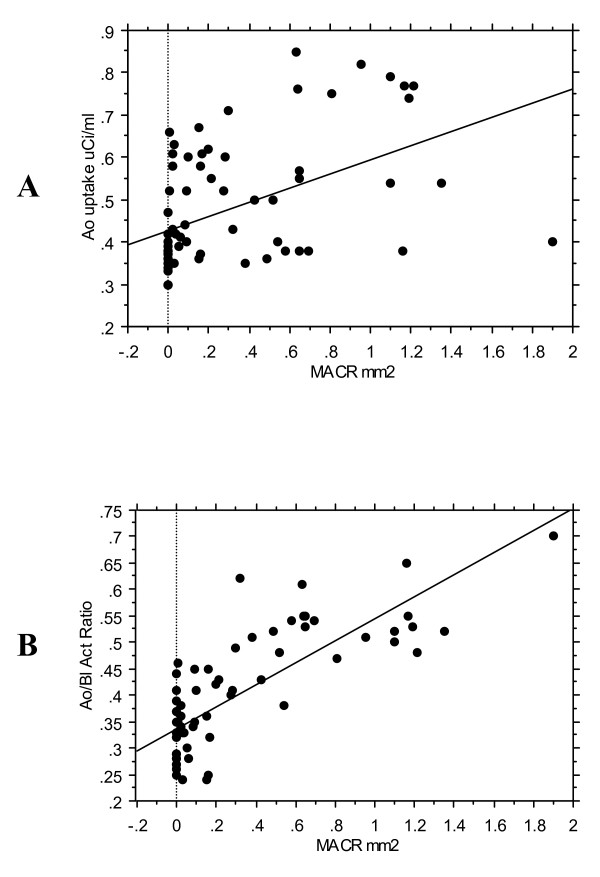
**The correlation of macrophage areas and FDG uptake**. Linear regression analyses showing the correlation of macrophage areas and FDG uptake in the segments (n = 72) of descending thoracic aorta of the rabbits, expressed as concentration (μCi/ml) (A) (r = 0.48, p < 0.0001) and ratio of aortic uptake-to-blood activity (B) (r = 0.80, p < 0.0001).

The correlation of aortic FDG uptake with macrophage areas as measured by histopathology was statistically significant but not high (r = 0.48, p < 0.0001)(Figure 4A).

### Macrophage areas and the ratio of aortic uptake-to-blood radioactivity

When FDG uptake was expressed as the ratio of aortic uptake-to-blood activity, a higher correlation (r = 0.80, p < 0.0001) between the macrophage areas and the FDG uptake ratio was observed (Figure 4B).

## Discussion

An important mechanism responsible for the sudden and unpredictable onset of acute thrombosis is plaque rupture [1]. The risk of rupture depends more on the plaque's composition rather than its size [1,3]. Rupture occurs preferentially in plaques containing a soft, lipid-rich core that is covered by a thin cap of fibrous tissue [3]. Compared with intact caps, the ruptured ones usually are thinner and contain less collagen, have fewer smooth muscle cells and are heavily infiltrated by macrophage foam cells [3,13]. These macrophages are activated, indicating ongoing inflammation at the site of plaque disruption [9]. Advances in our understanding of the cell biology of atherosclerosis have led the search for new imaging techniques that can provide information about plaque composition. Several imaging methods have been adapted to detect vulnerable atherosclerotic plaques [14]. However, they are unable to provide information on cell biologic evens that determine risk of plaque rupture. Different constituents of atherosclerosis, including radiolabeled lipoproteins, monocytes, smooth muscle cells, platelets, and fibrinogen have been used as potential plaque-imaging agents. Recently, radiolabeled annexin [15] and peptide [16] have also been studied to localize thrombus. However, these radiolabeled tracers and imaging technology need to be improved for *in vivo *imaging and characterization of atherosclerotic lesions.

Both deoxyglucose (DG) and FDG, which are analogs of glucose, compete with glucose for uptake into metabolically active cells. FDG has been shown to be a substrate for hexokinase [17], and is taken up into metabolically active cells but is not further metabolized. As FDG accumulates in the cells, its uptake is a measure of metabolic activity. FDG has been widely used to estimate glucose metabolism in heart, brain, and tumor tissue by PET. The mechanism of accumulation of this tracer within the malignant tissue is largely due to the enhanced rate of glucose utilization by neoplastic cells [18]. Owing to increased metabolic demand for glucose, hexokinase activity is increased. Recent work using micro autoradiography has demonstrated that macrophages, which are frequently found as part of granulation tissue around the tumor, often showed a higher uptake of FDG than even the tumor cells themselves [10]. Thus FDG might be used as a marker to quantify macrophages in atherosclerotic lesions, and PET might be used to predict the vulnerability of atherosclerosis.

Due to the unavailability of a PET/CT scanner at the time, this study was conducted using a dedicated brain PET scanner, MR imaging was used for the localization of the anatomical structures on the PET images and facilitated regional analyses of PET images with histological results. In our current study, in the control group there were no atherosclerotic lesions and no macrophages in the aortic wall. Normal rabbits did not show uptake above that of background levels. The combination of an atherogenic diet and balloon denudation of the aorta induced significant atherosclerosis.

Atherosclerotic aortas showed significantly higher uptake of FDG than normal aortas (Fig. 3). The atherosclerotic plaques in the aorta of rabbits were well visualized. The FDG uptake was not evenly distributed in the entire descending atherosclerotic aorta (Fig. 3B). High uptake was observed within the plaques, compared to uninjured areas. When FDG uptake was expressed as concentration, the correlation of aortic FDG uptake with macrophage areas as measured by histopathology was statistically significant but not high (r = 0.48, p < 0.0001) (Fig. 4A). It has been reported [19] that blood FDG activity is high compared to uptake in injured arterial segments in the experimental rabbit model. Because the present PET imaging was taken 30 min post-injection of FDG, the residual blood activity and FDG uptake in surrounding tissues might have impeded accurate quantification of aortic uptake. To reduce the influence of blood pool and surrounding structures, FDG uptake was expressed as the ratio of aortic uptake-to-blood activity, and aortic uptake was carefully measured from coregistered MR-PET images. We found that the ratio of aortic uptake-to-blood activity was better correlated to the macrophage areas assessed by histopathology (r = 0.80, p < 0.0001) (Fig. 4B). Delayed imaging, which allows more complete FDG clearance from blood, could provide additional benefits for the quantification of FDG uptake in aorta. Recent animal [20] and human FDG PET studies [21] of atherosclerotic plaque has confirmed that quantification of FDG uptake can be better achieved when performing imaging 210 min and 190 min post-injection of FDG, respectively.

This preliminary study shows that FDG PET detects and quantifies *in vivo *macrophage content within aortic atherosclerosis in an experimental rabbit model. Ledermanet et al. [19] and Rudd et al. [21] have showed that tritium-labeled glucose analogs accumulate in macrophage-rich atherosclerotic plaques in vitro. Ogawa et al. [20] recently investigated the relationship between the accumulation of FDG and the pathologic characteristics of aortic atherosclerotic lesions in Watanabe heritable hyperlipidemic (WHHL) rabbits. Despite the animal model being different from ours, they found that FDG uptake was well correlated with the number of macrophages. This is consistent with the finding in our studies, suggesting that macrophages are responsible for the accumulation of FDG in atherosclerotic lesions. Recently a clinical trial to detect atherosclerotic lesions by radionuclide imaging with FDG was also reported [21]. The study shows that plaque macrophage activity, the major determinant of plaque rupture, can be imaged by FDG PET. Together these and our studies suggest that FGD PET has the potential to monitor plaque inflammation *in vivo*, and to provide invaluable metabolic and composition information of the plaque. FGD PET then can assist in the stratification of patients at risk of plaque rupture and subsequent thrombosis. Furthermore, this promising non-invasive imaging technique might be used to assess serial progression and regression of macrophage content in different experimental models as well as in humans. Studies are currently under way to evaluate the potential of FDG PET for this application.

## Conclusion

PET FDG activity correlated with macrophage content within aortic atherosclerosis. This imaging approach might serve as a useful non-invasive imaging technique and potentially permit monitoring of relative changes in inflammation within the atherosclerotic lesion.

## Abbreviations

FDG: ^18^F-FDG

PET: Positron Emission Tomography

AALAC: American Association for the Accreditation of Laboratory Animal Care.

MR: Magnetic Resonance

## Competing interests

The author(s) declare that they have no competing interests.

## Authors' contributions

GH, SGW, AGZ, OJR, and JJB carried out animal model, histopathology and MR experiments, and participated in the design and discussion. ZZ, GF, SGW, CT, MSB and JM carried out PET studies and participated in the design and discussion. VF, JJB, ZZ, JM developed the initial concept and participated in the design and discussion. ZZ drafted the manuscript, JM edited the manuscript.

## Pre-publication history

The pre-publication history for this paper can be accessed here:


